# Efficacy of 42-month oral administration of glucoraphanin in preventing cognitive decline in individuals at elevated risk of dementia, including those with mild cognitive impairment: a randomized, double-blind, placebo-controlled pilot study

**DOI:** 10.3389/fnut.2026.1740494

**Published:** 2026-01-26

**Authors:** Sunao Shimizu, Shuya Kasai, Chieko Suzuki, Tomoya Kon, Hiroyuki Suganuma, Shigenori Suzuki, Koichi Murashita, Shigeyuki Nakaji, Kazushige Ihara, Masahiko Tomiyama, Ken Itoh

**Affiliations:** 1 Diet & Well-being Research Institute, KAGOME Co., Ltd., Nasushiobara, Japan; 2 Department of Vegetable Life Science, Hirosaki University Graduate School of Medicine, Hirosaki, Japan; 3 Department of Stress Response Science, Biomedical Research Center, Hirosaki University Graduate School of Medicine, Hirosaki, Japan; 4 Department of Neurology, Hirosaki University Graduate School of Medicine, Hirosaki, Japan; 5 Research Institute of Health Innovation, Hirosaki University, Hiroaki, Japan; 6 Department of Preemptive Medicine, Innovation Center for Health Promotion, Hirosaki University Graduate School of Medicine, Hirosaki, Japan; 7 Department of Social Medicine, Hirosaki University Graduate School of Medicine, Hirosaki, Japan

**Keywords:** Alzheimer’s disease, glucoraphanin, MCI screen, memory, memory performance index, mild cognitive impairment, prevention, sulforaphane

## Abstract

**Background:**

Nuclear factor erythroid 2-related factor 2 (Nrf2) is a transcription factor that regulates cellular defense mechanisms and has been proposed as a therapeutic target for Alzheimer’s disease (AD). Preclinical studies suggest that long-term oral administration of glucoraphanin (GLR), a natural Nrf2 activator, mitigates age-related cognitive decline in animal models.

**Objective:**

This study evaluated the long-term efficacy of GLR supplementation on cognitive function in older adults at an elevated risk for AD, including those with mild cognitive impairment (MCI).

**Methods:**

In a 42-month randomized, double-blind, placebo-controlled trial, 26 participants aged 63–90 years with memory impairment were randomly assigned to receive either 30 mg/day of GLR (*n* = 13) or placebo (*n* = 12). The primary outcome was the change in Memory Performance Index (MPI) scores from the MCI Screen. Secondary outcomes included conversion/reversion rates between normal cognition and MCI.

**Results:**

Ten participants in the GLR group and nine participants in the placebo group completed the trial. Analysis using a Linear Mixed Model (LMM) across the entire study period revealed a significant group by time-point interaction for MPI scores, with the GLR group showing a significantly greater improvement in MPI scores compared to the placebo (*p* = 0.012). No significant group difference was observed in the initial 6 months, but a marginal difference in favor of GLR appeared in the later phase (30 and 42 months), including the 42-month endpoint (*p* = 0.079). Conversion/reversion rates were not significantly different. The GLR group demonstrated superior performance on immediate recall and delayed free recall tests (*p* < 0.001 and *p* = 0.012, respectively). MCI participants showed a greater MPI improvement with GLR (*p* = 0.029). No severe adverse events related to the intervention were reported.

**Conclusion:**

Long-term GLR supplementation may help preserve cognitive function in individuals at elevated risk for AD, particularly those with MCI. Larger trials are warranted to confirm efficacy and clarify underlying mechanisms.

## 1 Introduction

Alzheimer’s disease (AD) is one of the most critical neurodegenerative disorders worldwide. According to the World Health Organization (WHO), dementia affected approximately 47 million people globally in 2015, with projections indicating increase to 75 million by 2030 and 132 million by 2050. AD is the most common form of dementia, according for 60–70% of cases (1). Japan is currently experiencing rapid population aging, with 29.3% of the population aged 65 or older and 16.8% aged 75 or older in 2024. Dementia, which progress with age, poses a significant public health challenge in Japan (2). Estimates from 2015 suggest that approximately 5 million Japanese individuals were living with dementia, and by 2045, the prevalence among individuals aged 65 years and over is expected to exceed 25% (3, 4). Despite extensive research, no definitive cure for AD has yet been established (5). While multifactorial interventions have shown promise in improving symptoms, current pharmacological treatments are limited (6). Symptomatic therapies include acetylcholinesterase inhibitors and antiglutamatergic agents, while disease-modifying treatments such as lecanemab and donanemab are effective only in the early stages of AD (5, 7–9). Given the lack of breakthrough therapeutic options, preventive strategies are of paramount importance in addressing the growing burden of AD.

It is widely accepted that AD progresses from a reversible state of mild cognitive impairment (MCI) to an irreversible state of dementia. Therefore, early detection and intervention at the MCI stage are crucial to preventing the onset of AD (10). Exercise therapy and several dietary interventions have been reported to be potentially effective in improving cognitive function in individuals with MCI (11, 12). However, the effectiveness of these approaches depends heavily on continuity and consistent practice. Consequently, it is essential to develop preventive strategies that are simple, practical, and sustainable over the long-term.

The amyloid and tau pathways have been confirmed as central mechanisms in AD, with amyloid precursor protein (APP) processing leading to amyloid peptides and plaques and tau protein aggregation resulting in neurofibrillary tangles. Crucially, the vascular, mitochondrial, and neuroinflammatory pathways also interact with these core mechanisms through one-way or reciprocal influences, contributing to neurodegeneration and disease progression (13). Mitochondrial dysfunction has, therefore, also gained attention as an important pathophysiological mechanism in AD. Mitochondrial impairment is implicated in various neurodegenerative diseases, including Parkinson’s disease, and has been reported in the brains of AD patients (14). For instance, the activity of mitochondrial respiratory chain complex IV (cytochrome c oxidase) is significantly reduced in the hippocampus of AD patients (15). Additionally, in mitochondrial cybrids generated by transfecting a human teratoma cell line (NT2) with mitochondrial DNA (mtDNA) from an AD patient, cytochrome c oxidase activity was decreased and reactive oxygen species (ROS) level were elevated compared to cybrids transfected with mtDNA from an age-matched healthy individual (16). Mitochondria naturally produce ROS during electron transfer through the mitochondrial respiratory chain (17). When mitochondrial function is impaired, excessive ROS are released, leading to oxidative damage in surrounding tissues.

Consequently, the present study focused on the transcription factor Nuclear Factor Erythroid 2-related factor 2 (Nrf2, gene name *Nfe2l2*), which play key roles in the antioxidant stress response and maintenance of mitochondrial function. Nrf2 regulates the expression of phase II detoxifying enzymes and antioxidant proteins, such as heme oxygenase-1 (HO-1), through its interaction with the antioxidant responsive element (ARE) (18). It also regulates genes involved in proteasome activity, autophagy, anti-inflammation, nerve growth factor signaling, and mitochondrial quality control. Therefore, Nrf2 is considered a promising target for the prevention of dementia. Indeed, Nrf2 activation has been shown to enhance cognitive function in various animal models (19). Certain cruciferous vegetables are rich in natural Nrf2 activators, with sulforaphane (SFN) being one of the most studied. SFN is a bioactive compound derived from glucoraphanin (GLR), which is abundant in broccoli sprouts (BS) (20). In plants, GLR is converted to SFN by the enzyme myrosinase, which is compartmentalized within plant cells and becomes active upon tissue disruption (e.g., grinding). In heat-extracted GLR preparations, plant-derived myrosinase is inactivated, and GLR is instead converted to SFN by bacterial myrosinase in the intestinal microbiota. However, it was confirmed that the supplementation of the diet with exogenous myrosinase via mustard powder increased the bioavailability of sulforaphane derived from broccoli containing thermally-inactivated myrosinase in healthy human subjects (21). This highlights the critical importance of co-administrating GLR with an active myrosinase source. Supporting this co-administration strategy, we previously demonstrated that combinational treatment with GLR and mustard (myrosinase) in mice significantly enhanced Nrf2 activation in the intestine and liver compared with GLR administration alone (22).

We previously demonstrated that the long-term administration of GLR combined with myrosinase in Senescence-Accelerated Mouse Prone 8 (SAMP8) mice, a model of age-related cognitive decline, preserved cognitive function, activated Nrf2, and enhanced mitochondrial biogenesis (22). Preliminary clinical studies have also reported improvement in symptoms of patients with schizophrenia and autism spectrum disorders following GLR supplementation (23). Furthermore, a 12-week supplementation of GLR improved age-related cognitive decline in healthy elderly individuals (24, 25). However, the efficacy of GLR in individuals with MCI, as well as its long-term cognitive protective effects remains unclear. Therefore, the present randomized controlled trial (RCT) was conducted to investigate the effects of GLR intake on the maintenance or improvement of cognitive function in individuals at elevated risk of developing dementia, including those with MCI.

## 2 Materials and methods

### 2.1 Design and setting of the randomized controlled trial

This RCT was conducted from February 2020 to July 2023 in Aomori, Japan. The study protocol was approved by the Ethics Committee of Hirosaki University General Certified Review Board (approval number: 2019-A-002). The trial was registered with the Japan Registry of Clinical Trials (jRCT, registered number: jRCTs021190008; registered on September 9, 2019; https://jrct.mhlw.go.jp/latest-detail/jRCTs021190008), and this report adheres to the CONSORT guidelines for reporting randomized trials. There was no participants or public involvement in the design, conduct, and reporting of this study. This study involved human volunteers and was conducted in accordance with the Declaration of Helsinki and the ethical guidelines for medical and biological research. Written informed consent was obtained from all participants prior to enrollment.

Cognitive function was assessed using the MCI-Screen (MCIS) test, a validated tool developed to detect MCI (26). The MCIS provides memory performance index (MPI) score. The primary outcome of this study was defined as the change in MPI score from baseline to the 42-month endpoint, assessed using repeated measures data from the GLR and placebo groups.

### 2.2 Participants

Participants were selected based on screening criteria indicating suspected memory impairment, identified during the memory assessments conducted as part of the Iwaki Health Promotion Project Medical Checkup between 2014 and 2017 (27). Eligible individuals met all of the following (1)–(3) criteria: (1) cognitive dysfunction recognized by the individual or its family; (2) delay recall score are less than 2 points on questionary 5 of Mini-Mental State Examination (MMSE); (3) scores on the Wechsler Memory Scale-Revised (WMS-R) Logical Memory Test II meet the following thresholds: 8 points or less for individuals with ≥16 years of education, 4 points or less for those with 10–15 years, and 2 points or less for those with ≤9 years of education. Among the screened individuals, those who provided informed consent, were not diagnosed with dementia, were aged 60 years or older, and were capable of participating in an exercise program were included in the study. Participants were excluded if they met any of the following criteria: (1) the presence of a serious illness (e.g., advanced cancer, diabetes, hypertension); (2) use of cholinesterase inhibitors for dementia treatment; (3) allergy to any ingredients in the experimental supplement; (4) presence of psychosis or psychiatric symptoms; or (5) diagnosis of dementia.

Prior to enrollment, baseline assessments were conducted for all enrolled participants, and each was screened for dementia or MCI. The enrolled participants were randomly assigned to either the GLR or placebo group, based on their MCI diagnosis. All participants were instructed to record their supplement intake, exercise time (in minutes), and physical condition on daily basis. These records were collected monthly, and average daily exercise time was calculated on a monthly basis for the purpose of analysis.

Cognitive assessments using the MCIS were conducted at baseline and at 3, 6, 18, 30, and 42 months post-intervention. Dementia and MCI diagnoses were reassessed at 18 and 42 months. After 18 month of intervention, urine samples were collected to measure sulforaphane N-acetyl-L-cysteine (SFN-NAC) levels, a urinary metabolite of GLR.

Dementia and MCI diagnosis at baseline, 18, and 42 months were determined according to Petersen’s criteria (28). Baseline evaluation also included screening for neurosurgical conditions such as chronic subdural hematoma and brain tumors to differentiate from MCI. Imaging was performed using 3 T magnetic response imaging (MRI) and analyzed with the Voxel-Based Specific Regional Analysis System for Alzheimer’s Disease (VSRAD, Eisai, Japan) at the Hirosaki University Hospital.

### 2.3 Sample size calculation

The required sample size was determined using EZR (R version 3.3.3, 2017). This study was designed as a pilot RCT primarily to assess the feasibility and preliminary efficacy of GLR intervention. Therefore, a formal sample size calculation for definitive statistical power was not performed. Instead, the sample size was determined based on practical considerations and previous literature (29, 30). A mean difference in the MPI Score between the groups was estimated to be 6 points, based on published data from a study investigating the effects of music therapy on MPI scores (unpublished) (29). The common standard deviation was estimated to be 5 points, derived from a cross-sectional study involving Japanese adults (30). Assuming a significance level (*α*) of 0.05 and a statistical power (1-*β*) of 0.95, the required sample size was determined to be 22 participants, with 11 in each group. To account for potential dropouts and protocol non-adherence, the initial sample size was set at 26 participants, with 13 in each group, but no additional participants were recruited to offset attrition due to the pilot nature of this study.

### 2.4 Randomization and allocation

Participants were randomly assigned to either the GLR group (*n* = 13) or the placebo group (*n* = 13) using stratified block random allocation. Stratification was based on sex (male/female) and MCI diagnosis, and randomization was performed using computer-generated random numbers. The allocation was managed by an independent allocation coordinator who was not involved in any other aspect of the study. An allocation table was created and concealed until the completion of data analysis. Except for the allocation coordinator, none of researchers involved in the trial were aware of the group assignments, ensuring blinding throughout the study.

### 2.5 Overview of the intervention

Participants allocated to the GLR or placebo groups received their respective supplements by mail once per month and were instructed to take three capsules daily for 42 months (from February 2020 to July 2023). The GLR supplement contained 30 mg of GLR for three capsules, while the placebo supplements contained no GLR. Both supplements were identical in color and size, ensuring blinding of participants to group assignment. To monitor adherence, participants were asked to return unused supplements by mail. Given the extended duration of the trial (42 months), a simple voluntary exercise program was offered to both groups to encourage continued participation and to address ethical considerations related to long-term intervention. Cognitive function was assessed using the MCIS at baseline and at 3, 6, 18, 30, and 42 months. Dementia and MCI diagnoses were evaluated at baseline, 18 months, and 42 months. Additionally, baseline assessments were conducted prior to intervention initiation. After 18 months of supplementation, urine samples were collected to measure SFN-NAC levels. An interim analysis was performed at 18 months. The trial was to be terminated if there was a significant difference in the change from baseline in MPI scores between groups at the 18-month time point.

### 2.6 Experimental supplements

Participants were instructed to take three capsules of either the GLR or placebo supplements daily for 42 months. The capsules were consumed with water in a single dose each day. The GLR supplement (Braphanin™, KAGOME CO., LTD., Nagoya, Japan) contained 30 mg of GLR purified from broccoli sprouts, along with 120 mg of mustard powder per three capsules. Mustard powder was included as a source of exogenous active myrosinase to enhance the enzymatic conversion of GLR to SFN (21). The placebo supplement contained 0 mg of GLR. Both supplements were identical in color and size to ensure blinding. KAGOME CO., LTD. (Nagoya, Japan) provided both the GLR and placebo supplements. Researchers, participants, and outcome assessors remained blinded to group allocation until the conclusion of the study.

### 2.7 Exercise program

All participants in both groups were offered a simple, voluntary exercise program consisting of stretching, recreational activities, and low-intensity aerobic exercise. Sessions were held at the Iwaki Welfare Center (Hirosaki, Japan). The program was initially scheduled to be conducted once per week for 1 hour during the first 6 months, and once every 2 months for 1 hour over the following 3 year. However, due to the outbreak of SARS-CoV-2 after the trial began, the actual implementation was limited. During the first month, sessions were conducted weekly as planned, but thereafter, they were held irregularly as part of exercise guidance sessions for the target participants. In practice, exercise sessions were held during weeks 1, 2, 3, 4, 96, 145, 162, 172, and 179.

### 2.8 Cognitive function measurement

To assess cognitive function, the participants underwent the MCIS test, from which MPI scores were derived. The MPI score is a quantitative measure ranging from 0 to 100, with higher scores indicating better cognitive performance. The scale includes three immediate- and one delayed-recall trials, similar to the Consortium to Establish a Registry for Alzheimer’s Disease (CERAD) and the Alzheimer’s Disease Assessment Scale-Cognitive Subscale (ADAS-Cog) 10-word recall tests (26).

However, the MPI score may offer greater sensitivity to early MCI compared to raw CERAD or ADAS recall scores, as it is a demographically adjusted composite index. It was developed using detailed diagnostic and medical data from Alzheimer’s Disease Centers funded by the U.S. National Institutes of Health (NIH) and the National Institute on Aging (NIA). The MPI provides a consistent index for evaluating cognitive function across a spectrum from healthy individuals to those with MCI, and enables objective, quantitative assessment without reliance on subjective judgment (26).

The MCIS comprises six tasks: (1) Three immediate recall trials of 10-word list, (2) Judgment of short-term recall, (3) Triadic comparison of animals, (4) Delayed free recall of 10-word list, (5) Delayed cued recognition of 10-word list items, and (6) Delayed free recall of items from triadic comparison (31). Details of each task are described below:

1. Three immediate recall trials of 10-word list:

Participants are given three trials to memorize 10 words by repeating them aloud, followed by immediate recall. This task assesses accuracy, serial position effect, word repetition/fabrication, reproduction patterns, and variations.

1. Judgment of short-term recall:

After task (1), participants are asked to estimate how many of the 10 words they expect to recall one time after a short delay.

1. Triadic comparison of animals:

After task (2), participants perform a brief interference task lasting 2–3 min, in which they select the odd item out from randomly presented sets of three animals. This task does not directly assess cognitive function.

1. Delayed free recall of 10-word list:

After task (3), participants attempt to freely recall the same 10-word list used in task (1).

1. Delayed cued recognition of 10-word list items:

After task (4), participants are shown 20 words (10 target words and 10 distractors) and asked to respond YES or NO to indicate whether each word was part of the original list. Responses are scored as correct or incorrect. The “Delayed cued recall–yes” score reflects the number of correctly recognized target words, while the “Delayed cued recall–no” score reflects the number of correctly rejected distractors.

1. Delayed free recall of items from triadic comparison:

After task (5), participants are asked to recall as many of the nine animals used in task (3) as possible. Since they were not instructed to memorize these animals, this task minimizes test-related anxiety compared to the word list recall.

Standardized scores are calculated for task (1), task (3), and task (4) in addition to raw scores. These standardized scores represent the number of standard deviations the participant’s performance deviates from the norm for their demographic peer group (adjusted for age, race, education, and gender). The scores obtained from each MCIS task reflect cognitive function, as shown in Table 1 (32). The MCIS has been validated for use in the Japanese population and is provided by MILLENNIA Corporation (Tokyo, Japan) (33).

**Table 1:** The components of the MCI-screen.

Task	Variable	Cognitive domain
(1)	Three immediate recall trials of 10-word list	Attention, working memory, comprehension
(2)	Judgment of short-term recall	Insight, awareness of cognitive abilities
(3)	Triadic comparison of animals	Working memory, judgment, semantic memory, comprehension
(4)	Delayed free recall of 10-word list	Short-term memory
(5)	Delayed cued recognition of 10-word list items	Recognition (information storage), source memory, comprehension, response bias
(6)	Delayed free recall of items from triadic comparison	Associative memory

### 2.9 Urine analysis

To assess whether SFN was absorbed into the body, the urine excretion level of SFN-NAC, a major metabolite of SFN, was analyzed in urine samples collected from participants 1–24 h after their final intake of either the GLR or placebo supplement at 18 months during the study period. The urine analysis was conducted according to previously reported methods (24, 25). Specifically, urine samples stored at −30 °C were thawed on ice and centrifuged (14,000 × g, 30 min, 4 °C) to precipitate proteins. The resulting supernatants were filtered through Ultracel-3 regenerated cellulose membrane for 3 kDa centrifugal filters (Merck Millipore, MA, USA). Filtrates (25 μL) along with the internal standard iberin (Tokyo Chemical Industry, Tokyo, Japan) and were subjected to HPLC-MS/MS analysis (a Shimadzu 20A HPLC system [Shimadzu, Kyoto, Japan] coupled to an LCQ Fleet electrospray ionization (ESI) ion trap mass spectrometer [Thermo Scientific, MA, USA] in duplicate). Quantification of SFN-NAC was performed using a five-point standard curve (Cayman Chemical Company, MI, USA), and the internal standard was dependent on the presence of two peaks monitored with the expected area ratio. Finally, the urinary levels of SFN-NAC were standardized to creatinine levels, which were determined using a commercial kit (Exocell, PA, USA) in accordance with the manufacturer’s instructions.

### 2.10 Statistical analysis

All statistical analyses were performed using the R software (Version 4.3.2). The Full Analysis Set (FAS) included 25 participants, derived from the 26 initially enrolled, after excluding one participant who withdrew post-randomization but prior to receiving the intervention. The Per-Protocol Set (PPS) was defined as all participants who successfully completed the entire study period. Between-group differences at each measurement point were analyzed using PPS by the Mann–Whitney U-test. Multivariate analysis using FAS to confirm the effects of GLR on the change of each cognitive function or the exercise time over the entire study period were performed by a linear mixed model (LMM) fitted to the following covariates: random effects for each individual; and fixed effects for categorical variables including group and group by time-point interactions; and fixed effects for continuous variables including baseline values, time-point and baseline value by time-point interactions. In this primary model, the time-point was treated as a continuous variable to evaluate the overall trend. As a post-hoc analysis to further investigate the intervention effects at each specific assessment point, an additional LMM was conducted by treating the time-point as a categorical variable. In this categorical model, fixed effects for categorical variables included the group, the time-point (as a factor), and the group by time-point interaction, while baseline values were included as a continuous covariate. This allowed for the estimation of standardized *β* (Std. *β*) coefficients, 95% confidence interval (95% CI), and *p* values for the interaction at each interval (3, 6, 18, 30, and 42 months). In the multivariate analysis of cognitive function, the results of the baseline assessment were used as baseline values. Since no exercise data were obtained before the trial, the multivariate analysis of exercise time was conducted using the value at first month after the trial as baseline. Multivariate analysis using FAS to confirm the effect of GLR on the raw value of each cognitive function over the entire study period was performed by a linear mixed model fitted to the following covariates: random effects for each individual; and fixed effects for categorical variables including group, and group by time-point interactions, continuous variables including time-point. The MPI score, the components of the MCIS, and time-point data were standardized and used in multivariate analysis. In multivariate analysis, statistical significance between the two groups was assessed based on group-time point interactions terms. The ratio of sex and MCI diagnosis at baseline, conversion rates (normal to MCI, MCI to dementia, and MCI to normal), and occurrences of adverse events were analyzed using the Fisher’s exact test. We initially planned to perform the Chi-squared test for the categorical data, but due to cells with an expected count of five or less in the resulting contingency table, the statistical method was changed to Fisher’s exact test. Subgroup analyses by sex or baseline diagnosis of MCI were performed as a post-hoc analysis. In all analyses, *p*-values < 0.05 were considered statistically significant.

## 3 Results

### 3.1 Study participants

**Figure 1:**
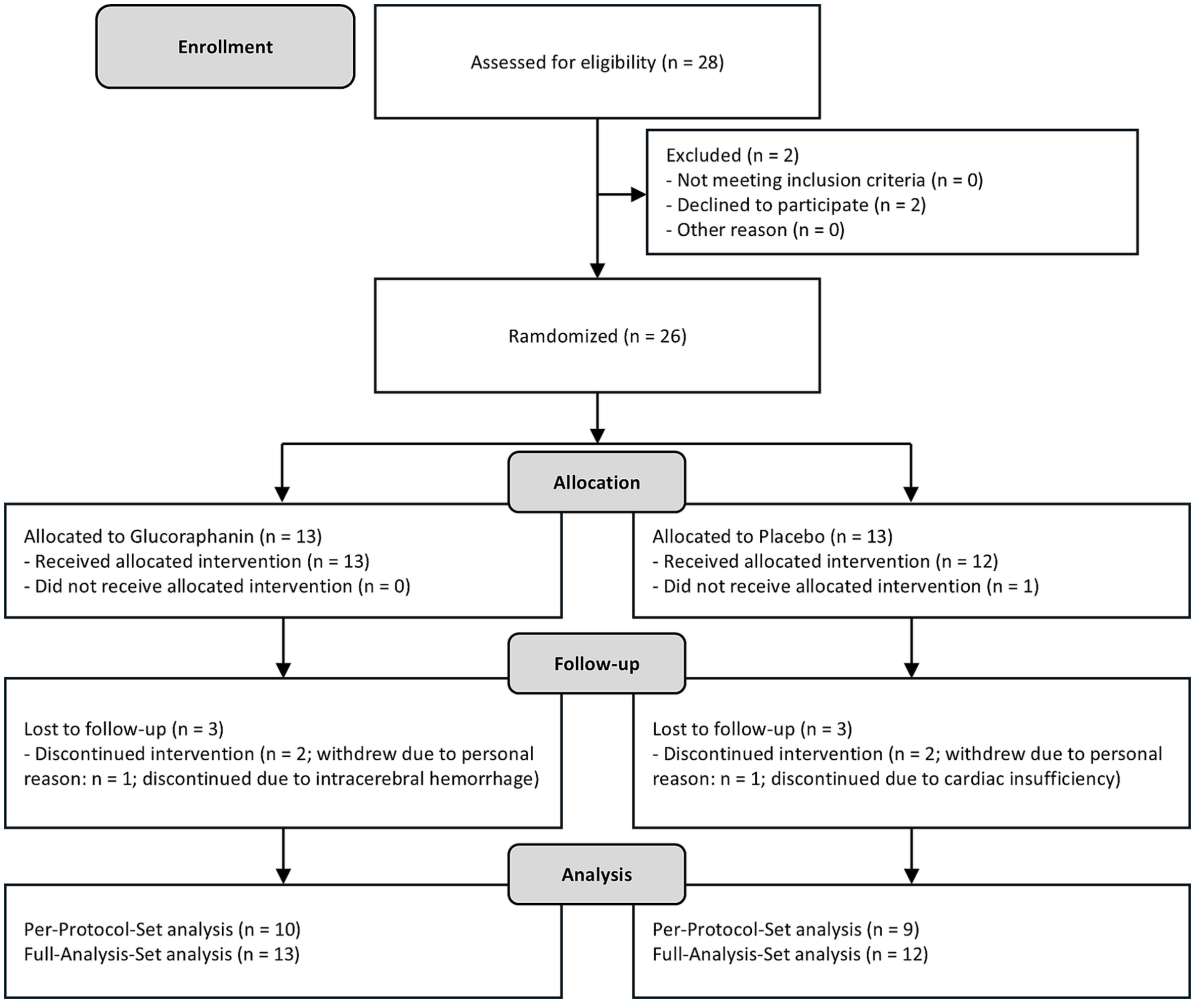
Flow diagram of the trial.

Figure 1 shows the flow diagram of participants throughout the RCT. A total of 28 individuals were enrolled at Hirosaki University Hospital in Japan between September 26 and December 26, 2019. Two participants withdrew prior to randomization, resulting in 26 individuals being assigned to either the GLR or placebo group, with 13 participants in each group. After allocation, one participant was excluded due to a pre-existing tumor diagnosis, which met the exclusion criteria. The intervention phase therefore began with 13 participants in the GLR group and 12 in the placebo group. The Full Analysis Set (FAS) was defined as these 25 participants (GLR: *n* = 13; Placebo: *n* = 12), adhering to the intention-to-treat principle. During the intervention, three participants in the GLR group discontinued: two withdrew for personal reasons, and one was withdrawn by the principal investigator due to intracerebral hemorrhage. In the placebo group, three participants also discontinued: two withdrew for personal reasons, and one was withdrawn due to cardiac insufficiency. At the endpoint evaluation, one participant was identified as a suspected case of dementia. However, the individual declined further diagnostic procedures required for confirmation and was managed as a suspected dementia case. The Per-Protocol Set (PPS) was defined as the participants who completed the entire 42-month study without major protocol deviations (GLR: *n* = 10; Placebo: *n* = 9).

Baseline characteristics, including age, sex ratio, MCI-to-normal ratio, and MPI score, did not differ significantly between the groups (Table 2). Among 13 participants who received GLR supplements, 10 completed the 42-month study. The mean ± standard deviations of adherence rate for the GLR supplements was 95.4 ± 1.9%. In the placebo group, 9 of 12 participants completed the 42-month study. The mean ± standard deviations of adherence rate for the placebo supplements was 94.3 ± 2.9%.

**Table 2:** Baseline characteristics.

Variable	Placebo group (*n* = 12)	GLR group (*n* = 13)	*p* value^a^
Female, *n* (%)	8 (66.7)	9 (69.2)	1.00
MCI, *n* (%)	9 (75)	9 (69.2)	1.00
Age in year	78 (72; 81)	72 (71; 78)	0.30
MPI score	54.3 (42.0; 56.2)	57.2 (46.1; 61.7)	0.41

Values are median (interquartile range) unless otherwise specified.

MPI, Memory performance Index.

a Fisher’s exact test for proportion and the Mann–Whitney U test for median.

**Figure 2:**
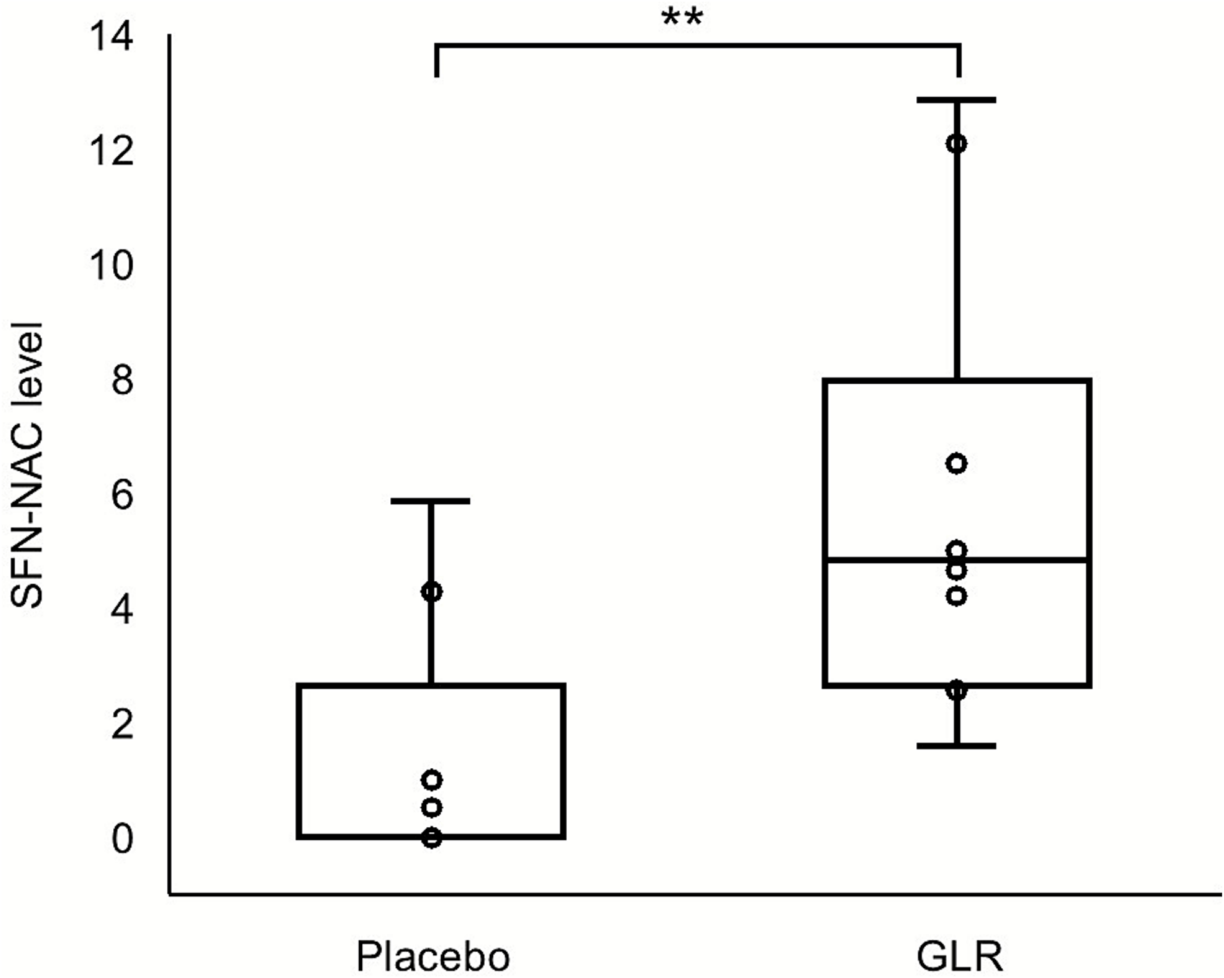
Urinary SFN-NAC levels at 18-month in the GLR and placebo group. Each data point represents the individual urinary SFN-NAC level measured 1–24 h after the final intake of the assigned supplement. The central bar indicates the median value; boxes represent the interquartile range (IQR); whiskers denote the minimum and maximum value; and crosses indicate the mean. Statistical comparison between groups was performed using Mann–Whitney *U* test. *p* < 0.01**.

Urinary SFN-NAC level at 19 months were significantly higher in the GLR group than in the placebo group (median [IQR (1Q; 3Q)] = 4.8 [3.1; 6.6] for the GLR group vs. 0.0 [0.0; 1.0] for the placebo group, *p* = 0.004. Figure 2). No significant difference was observed in daily exercise time between the groups at first month of intervention (median [IQR] (minutes) = 29.1 [11.4; 43.6] for the GLR group vs. 11.9 [5.2; 28.0] for the placebo group; *p* = 0.165, Mann-Whiteney U-test). However, the change in daily exercise time from baseline over the entire study period was significantly greater in the GLR group compared in the placebo group (Std. *β* [95% CI] = 0.15 [0.07, 0.23]; *p* < 0.001, linear mixed model).

### 3.2 Clinical efficacy

#### 3.2.1 Primary analysis

**Figure 3:**
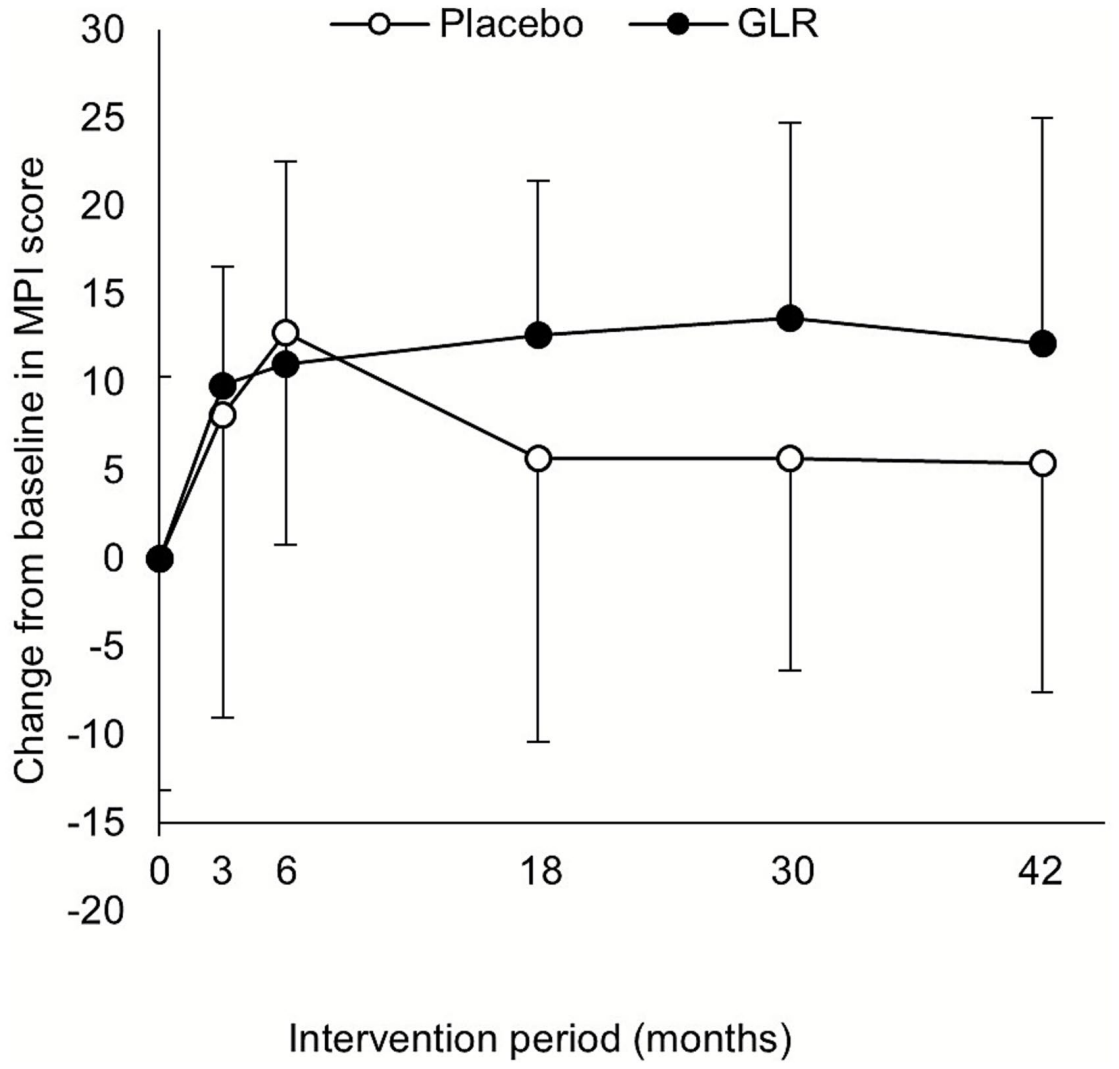
Mean MPI score change from baseline at month 3, 6, 18, 30, and 42. Data are presented as mean ± standard deviation (SD) based on the full-analysis set. The sample size (*n*) analyzed at each time point (month 0, 3, 6, 18, 30, and 42, respectively) was: GLR group (*n* = 13, 13, 12, 10, 10, and 10) and placebo group (*n* = 12, 12, 12, 12, 10, and 9).

Multivariate analysis using a LMM applied to the FAS revealed a significant group by time-point interaction for the change in MPI score from baseline over the entire 42-month period (Std. *β* [95% CI] = 0.29 [0.07; 0.51], *p* = 0.012). This indicates that the change in MPI scores from baseline in the GLR group was significantly greater compared to the placebo group over time (Table 3). In a post-hoc analysis treating time as a categorical variable to further explore this interaction at each assessment point, no significant group differences were observed in the early phase, specifically at 3 months (Std. *β* [95% CI] = 0.24 [−0.33; 0.8], *p* = 0.441) and 6 months (Std. *β* [95% CI] = 0.10 [−0.47; 0.66], *p* = 0.757). However, the effect of GLR became more pronounced over time, with a trend toward significance at 18 months (Std. *β* [95% CI] = 0.57 [−0.00; 1.15], *p* = 0.071) and significant group by time-point interactions emerging at 30 months (Std. *β* [95% CI] = 0.84 [0.24; 1.45], *p* = 0.012) and 42 months (Std. *β* [95% CI] = 0.71 [0.09; 1.33], *p* = 0.036, Supplementary Table 1). Consistent with these findings, point-in-time analysis showed the change in MPI score from baseline did not differ significantly between the two groups at the 3-month, 6-month, and 18-month assessments. However, the change in MPI score from baseline was numerically greater in the GLR group at the 30-month and 42-month endpoints. Specifically, the 42-month endpoint, the change in MPI score from baseline tended to be greater in the GLR group than in the placebo group; however, this difference did not reach statistical significance (Median [IQR] = 3.6 [2.3; 11.8] for the placebo group vs. 13.4 [8.4; 19.9] for GLR group. *p* = 0.079, PPS analysis, Table 3). In terms of raw value, the MPI score in the GLR group was significantly higher than that in the placebo group, both at endpoint (*p* = 0.028) and across all measurement points in the multivariate analysis (*p* = 0.022). At 3- and 6-month, MPI scores increased in both groups. However, while scores declined thereafter in the placebo group, they remained elevated in the GLR group (Table 3; Figure 3).

**Table 3:** Change from baseline and raw MPI scores over the study period.

Variable	Month	Placebo group			GLR group			Effect of GLR^a^^,^ ^b^			Placebo (*n* = 9)		GLR (*n* = 10)		Effect of GLR^c^
		*n*	Mean	SD	*n*	Mean	SD	Std.*β*	95% CI	*p* value^a^^,^ ^b^	Median	IQR	Median	IQR	*p* value^c^
Change from baseline	3	12	8.2	17.2	13	9.8	6.8	0.29	0.07; 0.51	0.012^a^*	9.3	6.2; 16.2	12.1	9.1; 14.8	0.720
	6	12	12.8	12.1	13	11.0	11.5				13.5	10.1; 16.2	12.7	5; 18.9	0.842
	18	12	5.7	16.1	12	12.7	8.7				5.0	3.4; 12.6	11.5	8.7; 18.9	0.278
	30	10	5.7	12.1	10	13.6	11.1				8.3	−0.7; 11.4	14.3	10.8; 16.7	0.079^†^
	42	9	5.4	12.9	10	12.2	12.8				3.6	2.3; 11.8	13.4	8.4; 19.9	0.079^†^
Raw value	0	12	49.3	13.1	13	53.8	10.3	0.25	0.04; 0.46	0.022^b^*	54.0	42.9; 55.2	55.2	46.3; 63.4	0.549
	3	12	57.4	17.2	13	63.6	6.8				60.5	57.3; 67.4	66.7	63.7; 69.3	0.182
	6	12	62.1	12.1	13	64.8	11.5				64.7	61.3; 67.4	67.3	59.6; 73.5	0.447
	18	12	54.9	16.1	12	66.5	8.7				56.2	54.6; 63.8	66.1	63.3; 73.5	0.113
	30	10	55.0	12.1	10	67.4	11.1				59.4	50.5; 62.6	68.8	65.4; 71.2	0.017*
	42	9	54.7	12.9	10	66.0	12.8				54.8	53.4; 63	68.0	63; 74.5	0.028*

GLR, Glucoraphanin; FAS, Full-Analysis-Set; PPS, Per-Protocol-Set; IQR; interquartile range showed as “1Q; 3Q.”

Standardized *β* (Std. *β*) value, 95% Confidence Interval (95% CI) and *p* value represent the group-by-time point interaction effects derived from multivariate analysis.

a Multivariate analysis using the FAS was conducted to assess the effect on the change from baseline throughout the entire study period. A linear mixed model was applied, incorporating random effects for individual participants and fixed effects for categorical variables (group and group by time-point interactions), as well as fixed effects for continuous variables (baseline values, time-point and baseline values by time-point interactions).

b Multivariate analysis using the FAS was conducted to assess the effect on the raw value throughout the entire study period. A linear mixed model was applied to the following covariates: random effects for individuals and fixed effects for categorical variables (group, and group by time-point interactions) and continuous variable (time-point).

c Data at each time point were analyzed using the PPS and compared between groups using the Mann–Whitney U test.

*p* < 0.05*, *p* < 0.1^†^.

**Figure 4:**
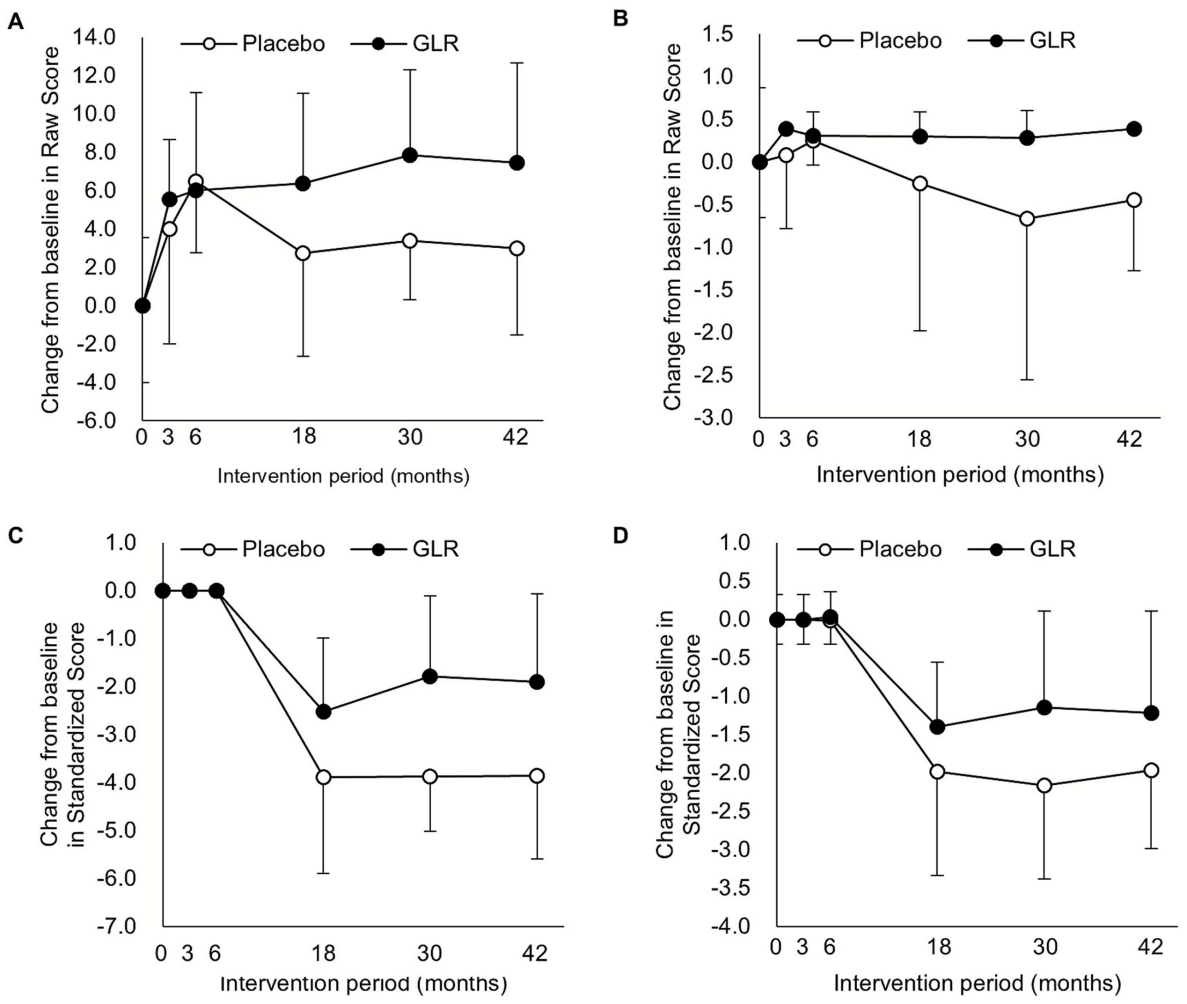
Mean change from baseline in some components of the MCI-Screen at months 3, 6, 18, 30, and 42. Each panel shows the mean change from baseline for: **(A)** the raw score of three immediate recall trials of a 10 word list; **(B)** the raw score of delayed cued recognition of 10 word list items; **(C)** the standardized score of three immediate recall trials of a 10 word list; and **(D)** the standardized score of delayed free recall of a 10 word list. Data are presented as the mean ± standard deviation (SD) based on the full-analysis set.

Analysis of MCIS components revealed that the GLR group had significantly higher performance than the placebo group in several domains. These included both raw and standardized scores for three immediate recall trials of 10-word list point, and the raw score for delayed cued recognition of 10-word list items (yes), and the standardized score in delayed free recall of 10-word list (Table 4; Figure 4). No significant differences were observed in the rate of reversion or conversion from basal status (Table 5).

**Table 4:** Effects of glucoraphanin supplementation on components of the MCI-Screen.

Components	Raw score			Standardized score		
	Std. *β*	95% CI	*p* value^a^	Std. *β*	95% CI	*p* value^a^
Three immediate recall trials of 10-word list point	0.41	0.14; 0.68	0.003**	0.43	0.21; 0.66	<0.001***
Judgment of short-term recall	0.21	−0.03; 0.44	0.086	-	-	-
Delayed free recall of 10-word list point	0.24	−0.01; 0.5	0.069	0.31	0.07; 0.54	0.012*
Delayed cued recognition of 10-word list items (yes)	0.38	0.07; 0.68	0.015*	0.27	−0.04; 0.55	0.081
Delayed cued recognition of 10-word list items (no)	−0.12	−0.46; 0.21	0.473	−0.14	−0.44; 0.17	0.387
Delayed free recall of items from triadic comparison	−0.19	−0.41; 0.02	0.083	-	-	-

Standardized *β* (Std. *β*) value, 95% Confidence Interval (95% CI) and p value were indicated as the group-time point interaction in multivariate analysis.

a Multivariate analysis using the FAS was conducted to assess the effect on the change from baseline throughout the entire study period. A linear mixed model was applied, incorporating random effects for individual participants and fixed effects for categorical variables (group and group by time-point interactions), as well as fixed effects for continuous variables (baseline values, time-point and baseline values by time-point interactions).

- Standardized Scores are not calculated for Judgment of short-term recall task and Delayed free recall of items from triadic comparison task, as they are not considered meaningful for these components.

*p* < 0.001***, < 0.01**, < 0.05*.

**Table 5:** Rates of reversion and conversion rate from baseline diagnosis.

Variable	Month	Placebo group (*n* = 9)	GLR group (*n* = 10)	*p* value^a^
State, *n* (%)				
Normal cognitive	0	3 (33.3%)	4 (40%)	1.000
	18	6 (66.7%)	8 (80%)	0.629
	42	6 (66.7%)	8 (80%)	0.629
MCI	0	6 (66.7%)	6 (60%)	1.000
	18	3 (33.3%)	2 (20%)	0.629
	42	2 (22.2%)	2 (20%)	1.000
Dementia (suspected) ^b^	0	0 (0%)	0 (0%)	1.000
	18	0 (0%)	0 (0%)	1.000
	42	1 (11.1%)	0 (0%)	0.474
Reversion and conversion rate, % (after state)				
MCI to Normal (MCI/Normal)	18	66.7% (4/2)	66.7% (4/2)	1.000
	42	66.7% (4/2)	66.7% (4/2)	1.000
Normal to MCI (Normal/MCI)	18	33.3% (1/2)	0% (0/4)	0.429
	42	33.3% (1/2)	0% (0/4)	0.429
MCI to Dementia (Dementia/MCI)	18	0% (0/6)	0% (0/6)	1.000
	42	16.7% (1/5)	0% (0/6)	1.000

Values are presented as median (interquartile range), unless otherwise specified.

MCI, Mild cognitive impairment.

- Not calculable.

a Reversion and conversion rate at each time point were analyzed using the Fisher’s exact test.

b The case was classified as suspected dementia due to refusal of definitive diagnosis.

*p* < 0.001***, < 0.01**, < 0.05*.

#### 3.2.2 Subgroup analysis by basal state in MCI diagnosis

Participants were stratified into MCI and cognitively normal groups based on their baseline diagnosis, and the effects of GLR were evaluated accordingly (Table 6; Supplementary Table 2). Among participants diagnosed with MCI at baseline, the change in MPI score from baseline was significantly greater than that of the placebo group, both over the entire study period and at the 42-month endpoint (*p* = 0.029 and *p* = 0.015, respectively; Table 6). In contrast, among cognitively normal participants, the MPI score in the GLR group remained consistently higher than that in the placebo group throughout the study period; however, the difference did not reach statistical significance (*p* = 0.281, Supplementary Table 2).

**Table 6:** Changes from baseline and raw values of MPI scores in participants diagnosed with MCI.

Variable	Month	Placebo			GLR			Effect of GLR^a^^,^ ^b^			Placebo (*n* = 6)		GLR (*n* = 6)		Effect of GLR^c^
		n	Mean	SD	n	Mean	SD	Std. *β*	95% CI	*p* value^a^^,^ ^b^	Median	IQR	Median	IQR	*p* value^c^
Change from baseline	3	9	8.6	19.6	9	10.2	5.2	0.32	0.04; 0.6	0.029^a^*	10.8	6.5; 17	11.7	10.6; 13.9	0.240
	6	9	14.2	13.9	9	11.6	10.5				15.6	11.2; 19	13.7	6.7; 18.5	0.394
	18	9	6.4	18.0	8	13.0	6.5				11.2	6.4; 15.1	10.5	8.3; 17.6	0.240
	30	7	7.8	13.9	6	14.6	3.0				11.1	4.1; 16.7	13.9	12.4; 15.4	0.065^c†^
	42	6	5.0	15.1	6	14.7	4.9				6.0	1.1; 10.7	13.1	9.4; 17.1	0.015^c^*
Raw value	0	9	48.6	11.1	9	55.0	11.7	0.26	0.00; 0.52	0.051^b†^	47.3	40.3; 54.3	55.8	46.3; 65.0	0.937
	3	9	57.8	11.4	9	66.3	4.7				59.4	55.1; 65.7	66.7	65.5; 68.9	0.937
	6	9	58.4	16.1	9	68.0	9.3				64.2	59.9; 67.6	68.6	61.6; 73.5	0.818
	18	9	58.2	14.6	8	67.9	7.2				59.8	55.0; 63.7	65.4	63.3; 72.6	0.818
	30	7	55.8	14.9	6	68.3	3.0				59.8	52.7; 65.4	68.8	67.3; 70.3	0.589
	42	6	51.9	15.1	6	68.4	4.9				54.6	49.8; 59.3	68.0	64.4; 72.0	0.180

GLR, Glucoraphanin; FAS, Full-Analysis-Set; PPS, Per-Protocol-Set; IQR; interquartile range showed as “1Q; 3Q.”

Standardized *β* (Std. *β*) value, 95% Confidence Interval (95% CI) and *p* value represent the group-by-time point interaction effects derived from multivariate analysis.

a Multivariate analysis using the FAS was conducted to assess the effect on the change from baseline throughout the entire study period. A linear mixed model was applied, incorporating random effects for individual participants and fixed effects for categorical variables (group and group by time-point interactions), as well as fixed effects for continuous variables (baseline values, time point and baseline values by time-point interactions).

b Multivariate analysis using the FAS was conducted to assess the effect on the raw value throughout the entire study period. A linear mixed model was applied to the following covariates: random effects for individuals and fixed effects for categorical variables (group, and group by time-point interactions) and continuous variable (time-point).

c Data at each time point were analyzed using the PPS and compared between groups using the Mann–Whitney U test.

*p* < 0.05*, *p* < 0.1^†^.

#### 3.2.3 Subgroup analysis by sex

Subgroup analysis stratified by sex revealed trends consistent with those observed in the overall participant analysis. In both female and male participants, the GLR group demonstrated a significantly greater change in MPI scores from baseline over the entire study period compared to the placebo group (*p* = 0.034 for the female; *p* = 0.045 for the male). At the 42-month endpoint, MPI scores in the GLR group remained higher than those in the placebo group for both sexes; however, these differences were not statistically significant (Supplementary Table 3).

### 3.3 Clinical safety

Table 7 summarizes adverse events observed during the study. There was no notable and significant difference in the incidence of adverse events between the GLR and placebo groups.

**Table 7:** List of reported adverse events.

Adverse events	Events/ individual presenting with symptoms		*p* value^a^
	Placebo group (*n* = 12)	GLR group (*n* = 13)	
Neoplasms			
Gastric cancer	1/1	0/0	0.480
Eye			
Cataractous	0/0	1/1	1.000
Circulatory system			
Cardiac insufficiency	1/1	0/0	0.480
Intracerebral hemorrhage	0/0	1/1	1.000
Colitis	0/0	1/1	1.000
Constipation	0/0	2/1	1.000
Diarrhea	0/0	2/2	0.480
Dyspepsia	1/1	5/3	0.593
Foodborne disease	0/0	1/1	1.000
Vomit	0/0	1/1	1.000
Skin and subcutaneous tissue			
Urticaria	0/0	1/1	1.000
Zoster	2/1	0/0	0.480
Musculoskeletal system			
Ankle fracture	1/1	0/0	0.480
Gouty	0/0	1/1	
lumbar disc herniation	1/1	0/0	0.480
Spinal stenosis	1/1	0/0	0.480
Genitourinary system			
Urinary infection	3/2	0/0	0.220
Urolithiasis	0/0	1/1	1.000
Symptoms and signs			
Arthralgia	0/0	1/1	1.000
Headache	0/0	1/1	1.000
Knee pain	2/2	2/2	1.000
Pruritus	0/0	2/2	0.480
Shoulder pain	0/0	5/2	0.480
Sore throat	0/0	1/1	1.000
Injury			
Abrasion	0/0	1/1	1.000
Bruise	2/2	1/1	0.593
Rib fracture	0/0	1/1	1.000
Stab wound	1/1	0/0	0.480

a Data were analyzed using the Fisher’s exact test.

## 4 Discussion

To our knowledge, this is the first study to investigate the long-term effects of GLR on cognitive function in individuals at elevated risk of developing dementia, including those diagnosed with MCI. In the present study, multivariate analysis demonstrated that the change in MPI score from baseline over the entire period was significantly greater in the GLR group compared to the placebo group. At the 42-month endpoint, the change in MPI score from baseline in the GLR group was nearly significantly higher than in the placebo group. In the short term, MPI scores increased in both groups; however, while scores declined over time in the placebo group, they remained elevated in the GLR group. In the placebo group, scores for “Three immediate recall trials,” “Delayed free recall,” and “Delayed cued recognition,” declined from baseline, whereas this decline was significantly inhibited in the GLR group. These findings suggest that GLR intake may help prevent progressive cognitive decline in individuals with MCI. Previous studies have reported that 12 weeks of GLR supplementation improved cognitive function in healthy elderly people (24, 25). Taken together, these results suggest that GLR may not only enhance cognitive function in the short term but also contribute to the long-term prevention of cognitive decline.

In the present study, MPI score increased in both the GLR and placebo group during the short-term period (3- and 6-month). However, there was no significant difference between the two groups. Aerobic exercise has been suggested to be a beneficial strategy for the treatment and prevention of MCI (11). The previous study reported that a 12-month intervention involving exercise training programs and nutritional lectures led to increased MPI scores in individuals with MCI (34). These findings suggest that short-term improvements in MPI score observed in both groups may be attributed to common factors such as physical activity, familiarity with the testing procedure, and the placebo effect in the placebo group. Furthermore, lifestyle modifications at the beginning of the trial, including the introduction of physical activity shared by both groups, may have had a predominant impact on cognitive performance. This common improvement likely masked the specific preventive effects of GLR on MPI scores during the early phase of the intervention.

In subgroup analysis based on baseline status in MCI diagnosis, participants with MCI in the GLR group showed a greater change in MPI scores from baseline both over the entire study period and at the endpoint, compared to those in the placebo group. Previous studies have reported that GLR intake improves cognitive function in healthy elderly people (24, 25). These findings suggest that GLR intake may help maintain cognitive function not only in healthy people but also in those diagnosed with MCI. Additionally, the subgroup analysis by sex revealed that the change in MPI score from baseline over the entire period was greater in the GLR group than the placebo group for both male and female participants. This indicates that GLR intake may be effective in preventing cognitive decline, regardless of sex.

In the present study, the GLR group performed significantly better than the placebo group in the “Three immediate recall trials,” “Delayed free recall,” and “Delayed cued recognition,” which are components of the MCIS. Each component reflects distinct aspects of cognitive function: immediate recall trial reflects attention, working memory, comprehension; delayed free recall reflects, short-term memory; and delayed cued recognition reflects recognition (information storage), source memory, comprehension, and response bias. Previous studies have shown that GLR intake can enhance processing speed performance and working memory in healthy elder adults (24, 25). Furthermore, our previous *in vivo* study demonstrated that GLR improved long-term memory in SAMP8 (22). Other *in vivo* studies have shown that SFN, the active form of GLR, ameliorates memory deficits in triple transgenic mouse model of AD (35), and suppresses impairment in spatial learning and memory in models administered lipopolysaccharide (LPS) (36), okadaic acid (37), scopolamine (38), and streptozotocin (39). These present and previous findings suggest that glucoraphanin contributes to the maintenance and improvement of memory. Since dementia and MCI are characterized by long-term progression, preventive strategies must have sustained effects. Although, several studies have reported cognitive benefits of dietary interventions in individuals with MCI, only a few have demonstrated long-term effects over 2 years or more, such as those involving docosahexaenoic acid (40), vitamin B (41), and virgin olive oil (42). The identification of GLR as a dietary component with long-term cognitive benefits represents a promising advancement in the prevention and management of MCI.

SFN, the active form of GLR, has been confirmed to possess antioxidant and anti-inflammatory properties via the Nrf2-Keap1 pathway (43). In AD pathology, the accumulation of amyloid-beta and tau proteins is thought to reduce Nrf2 levels, thereby weakening the antioxidant response. The reduction in Nrf2 further promotes the accumulation of amyloid-beta and tau by impairing their autophagy-mediated clearance. Consequently, Nrf2 activation is considered a potential approach to mitigate AD pathology (44, 45). Furthermore, our previous *in vivo* study demonstrated that long-term GLR intake suppressed age-related cognitive decline in SAMP8 mice and upregulated genes related mitochondrial biogenesis in the hippocampus (22). In the present study, MCIS components associated with the prefrontal cortex and hippocampal (32) were significantly higher in the GLR group compared to the placebo group. These findings suggest that GLR intake may suppress cognitive decline through mechanisms such as Nrf2 activation and enhancement of mitochondrial biogenesis in brain regions like the hippocampus. At the beginning of the intervention, there was no difference in daily exercise time between the two groups. However, over the course of the intervention, the GLR group exhibited significantly greater exercise time compared to the placebo group. SFN has been shown to benefit skeletal muscle health. For instance, Moradi, N., et al. reported that SFN increase mitochondrial respiration and ATP production in C2C12 myotubes cell (46). Bose, C., et al. demonstrated that SFN restored Nrf2 activity, mitochondrial function, cardiac function, and activation/differentiation of skeletal muscle satellite cells in aged C57BL/6 mice (47). Komine, S., et al. found that GLR intake ameliorated exercise-induced muscle soreness and damage (48). Additionally, a previous study showed GLR intake improve processing speed and reduced negative mood in healthy elderly individuals, as assessed by POMS test (25). Since negative mood has been reported to negatively correlate with physical activity (49, 50), GLR may have enhanced physical activity by promoting positive mood. Therefore, in the present study, GLR intake may have increased physical activity through both maintenance and improvement of skeletal muscle function and mood enhancement. Daily physical activity is known to improve cognitive function, suggesting that GLR may help prevent cognitive decline not only through direct effects on brain tissue but also via indirect effects mediated by increasing physical activity (51, 52). However, it cannot be ruled out that increased exercise time in GLR group occurred by chance during the intervention period. This possibility is raised by the voluntary nature of the provided exercise program, which likely led to inconsistent frequency and intensity of exercise both between and within individuals, a situation exacerbated by the small sample size. Moreover, the extent to which this change in exercise time may have mediated the effect on cognitive function remains unknown. Accordingly, further research is needed to clarify the relationship between GLR intake and physical activity. Glucoraphanin possess antioxidative and anti-inflammatory properties, and its health benefits have been demonstrated in numerous clinical trials across various diseases (53). For example, GLR intake has been reported to improve conditions such as fatty liver, diabetes, and dyslipidemia, all of which are known risk factors of AD (54–59). Moreover, multifactorial interventions have been shown to improve dementia and MCI, suggesting that GLR may contribute to cognitive health by supporting overall physiological well-being.

The results of the present study indicate that 30 mg/day of GLR over 42 months did not lead to severe side effects. This finding is consistent with previous studies in which 30 mg/day of GLR was consumed for 12 weeks without adverse effects (24, 25). Notably, the duration of the present trail was significantly longer than previous trials. A prior clinical trial also reported no abnormal clinical chemistry values in blood test following daily consumption of a broccoli sprout extract beverage containing approximately 268 mg/d of GLR for 12 weeks (60). To our knowledge, this is the first study to demonstrate that the long-term intake of GLR (42 months) may be beneficial for brain health without safety concerns. The characteristics of GLR, including its safety, suitability for long-term use, and broad health benefits, make it an ideal candidate as preventive nutrient for aging. The widespread consumption of GLR is expected to contribute to extending the healthy lifespan and improving public health outcomes.

### 4.1 Limitation

The present study has several limitations. First, the sample size was small. At the endpoint (42-month), only a significant trend in MPI score was observed, and a larger sample size is required to obtain robust results demonstrating the effect on MPI score. The small sample size, coupled with the lack of detailed baseline assessment of physical function and the absence of a detailed, longitudinal assessment of lifestyle factors (e.g., dietary habits) throughout the intervention period, precluded the determination of the effectiveness of the intervention on MCI conversion and reversion, or to assess the influence of age, daily exercise time, dietary habits and other background factors. Statistical adjustment for potential confounding effects, such as changes in background factors including exercise time during the intervention period, is inherently difficult in studies with such a small sample size.

Second, most of the study outcomes rely on subjective scales. Although the MCI screen, derived from the highly validated 10-word recall test, was considered an appropriate primary outcome measure for individuals with MCI, the absence of objective indicators prevents us from fully corroborating the results.

Third, since the participants included only individuals with normal cognition and MCI, it remains unclear whether similar effects would be observed in patients with dementia.

Fourth, as biomarkers and objective cognitive function indicators (such as electroencephalography [EEG] and eye tracking) were not evaluated, the molecular biological mechanisms and specific changes in brain activity by which GLR suppressed cognitive decline could not be elucidated in detail.

To address these limitations, future studies should be conducted on a larger scale, encompassing a detailed assessment of background factors, and include simultaneous assessment of biomarker changes and objective cognitive function indicators, thereby yielding more robust findings and contributing significantly to the elucidation of the underlying mechanisms.

## 5 Conclusion

In conclusion, the results of the present study suggest that the daily intake of GLR supplements has the potential to safely prevent cognitive decline in individuals at elevated risk for AD, particularly those with MCI. However, further clinical studies are needed to confirm the detailed effects and elucidate the mechanisms by which GLR supports brain health.

## Data Availability

The datasets presented in this article are not readily available due the ethical concerns but are available from the Hirosaki University COI Program Institutional Data Access / Ethics Committee for researchers who meet the criteria for access to the data. Researchers need to be approved by research ethics review board at the organization of their affiliation. Requests to access the datasets should be directed to the Hirosaki University COI Program Institutional Data Access / Ethics Committee, coi@hirosaki-u.ac.jp.
