# Supplementary material for: Efficacy of 42-month oral administration of glucoraphanin in preventing cognitive decline in individuals at elevated risk of dementia, including those with mild cognitive impairment: a randomized, double-blind, placebo-controlled pilot study
Source: Front Nutr. 2026 Jan 26;13:1740494. doi: 10.3389/fnut.2026.1740494 (PMC12884063; doi:10.3389/fnut.2026.1740494)
Supplement: Supplementary file 1 [file Data_Sheet_1.docx]

| **Supplemental table 1** Fixed Effects of Change from Baseline in MPI Score at Each Time Point | | | | | | |
| --- | --- | --- | --- | --- | --- | --- |
|  | Sample size | |  | Effect of GLR | | |
| Month | Placebo | GLR |  | Std.β | 95% CI | *P* value^a^ |
| 3 | 12 | 13 |  | 0.24 | -0.33; 0.8 | 0.441 |
| 6 | 12 | 13 |  | 0.10 | -0.47; 0.66 | 0.757 |
| 18 | 12 | 12 |  | 0.57 | -0.00; 1.15 | 0.071^†^ |
| 30 | 10 | 10 |  | 0.84 | 0.24; 1.45 | 0.012* |
| 42 | 9 | 10 |  | 0.71 | 0.09; 1.33 | 0.036* |
| Abbreviations: GLR, Glucoraphanin; FAS, Full-Analysis-Set; IQR; interquartile range showed as “1Q; 3Q”.  Standardized β (Std. β) value, 95% Confidence Interval (95% CI) and p value represent the group-by-time point interaction effects derived from multivariate analysis.  ^a^ Post-hoc multivariate analysis using the FAS was conducted to assess the effect on the change from baseline throughout the entire study period. A linear mixed model was applied, incorporating random effects for individual participants and fixed effects for categorical variables (group, time-point, group by time-point interactions, and baseline values by time-point interactions), as well as fixed effects for continuous variable (baseline values).  *p* < 0.05*, *p* < 0.1^†^. | | | | | | |

| **Supplemental table 2** Change from baseline and raw values of MPI score in cognitively normal participants | | | | | | | | | | | | | | | | | | | | | |
| --- | --- | --- | --- | --- | --- | --- | --- | --- | --- | --- | --- | --- | --- | --- | --- | --- | --- | --- | --- | --- | --- |
| Variable |  |  | Placebo | | |  | GLR | | |  | Effect of GLR^a, b^ | | |  | Placebo (n = 3) | |  | GLR (n = 4) | |  | Effect of GLR^c^ |
|  | Month |  | n | Mean | SD |  | n | Mean | SD |  | Std.β | 95% CI | *P* value^a, b^ |  | Median | IQR |  | Median | IQR |  | *P* value^c^ |
| Change from baseline | 3 |  | 3 | 7.0 | 5.3 |  | 4 | 9.0 | 10.5 |  | 0.22 | -0.17; 0.61 | 0.281^a^ |  | 8.8 | 4.9; 10.0 |  | 12.0 | 5.4; 15.6 |  | 0.629 |
|  | 6 |  | 3 | 8.9 | 2.0 |  | 4 | 9.9 | 15.2 |  |  |  |  |  | 8.4 | 7.8; 9.8 |  | 10.4 | 1.9; 18.4 |  | 1.000 |
|  | 18 |  | 3 | 3.6 | 8.6 |  | 4 | 12.0 | 13.5 |  |  |  |  |  | -0.1 | -1.3; 6.7 |  | 13.8 | 6.7; 19.2 |  | 0.629 |
|  | 30 |  | 3 | -0.8 | 8.9 |  | 4 | 12.1 | 18.7 |  |  |  |  |  | 2.0 | -4.4; 4.1 |  | 18.0 | 4.7; 25.4 |  | 0.400 |
|  | 42 |  | 3 | 3.8 | 5.8 |  | 4 | 8.4 | 20.6 |  |  |  |  |  | 6.7 | 2.0; 7.1 |  | 14.0 | -0.4; 22.8 |  | 0.857 |
|  |  |  |  |  |  |  |  |  |  |  |  |  |  |  |  |  |  |  |  |  |  |
| Raw value | 0 |  | 3 | 56.3 | 3.4 |  | 4 | 54.0 | 7.0 |  | 0.26 | -0.13; 0.65 | 0.196^b^ |  | 54.6 | 54.3; 57.4 |  | 55.2 | 49.5; 59.7 |  | 0.629 |
|  | 3 |  | 3 | 63.3 | 5.3 |  | 4 | 63.0 | 10.5 |  |  |  |  |  | 65.0 | 61.2; 66.3 |  | 66.0 | 59.4; 69.6 |  | 0.857 |
|  | 6 |  | 3 | 65.1 | 2.0 |  | 4 | 63.9 | 15.2 |  |  |  |  |  | 64.7 | 64.0; 66.0 |  | 64.4 | 55.9; 72.4 |  | 1.000 |
|  | 18 |  | 3 | 59.9 | 8.6 |  | 4 | 66.0 | 13.5 |  |  |  |  |  | 56.2 | 55.0; 62.9 |  | 67.9 | 60.7; 73.2 |  | 0.629 |
|  | 30 |  | 3 | 55.5 | 8.9 |  | 4 | 66.1 | 18.7 |  |  |  |  |  | 58.3 | 51.9; 60.4 |  | 72.0 | 58.7; 79.4 |  | 0.400 |
|  | 42 |  | 3 | 60.1 | 5.8 |  | 4 | 62.4 | 20.6 |  |  |  |  |  | 63.0 | 58.2; 63.4 |  | 68.1 | 53.7; 76.8 |  | 0.857 |
| GLR, Glucoraphanin; FAS, Full-Analysis-Set; PPS, Per-Protocol-Set; IQR; interquartile range showed as “1Q; 3Q”.  Standardized β (Std. β) value, 95% Confidence Interval (95% CI) and *p* value were indicated as the group-time point interaction in multivariate analysis.  ^a^ Multivariate analysis with FAS to confirm the effect for the mean difference throughout the entire study period was performed by a linear mixed model fitted to the following covariates: random effects for each personal; and fixed effects for categorical variables including group (0: placebo/1: GLR group) and group by time-point interactions; and fixed effects for continuous variables including baseline values, time-point and baseline values by time-point interactions  ^b^ Multivariate analysis with FAS to confirm the effect for the raw value throughout the entire study period was performed by a linear mixed model fitted to the following covariates: random effects for each personal; and fixed effects for categorical variables including group, and group by time-point interactions, continuous variables including time-point.  ^c^ The data for each time-point was analyzed with PPS using the Mann-Whitney U test. | | | | | | | | | | | | | | | | | | | | | |

| **Supplemental table 3** Change from baseline and raw values of MPI score in female and male participants | | | | | | | | | | | | | | | | | | | | | | | |
| --- | --- | --- | --- | --- | --- | --- | --- | --- | --- | --- | --- | --- | --- | --- | --- | --- | --- | --- | --- | --- | --- | --- | --- |
|  |  |  |  | Placebo | | |  | GLR | | |  | Effect of GLR^a, b^ | | |  | Placebo (n = 6) | |  | GLR (n = 8) | |  | Effect of GLR^c^ |  |
| Sex | Variable | Month |  | n | Mean | SD |  | n | Mean | SD |  | Std.β | 95% CI | *P* value^a, b^ |  | Median | IQR |  | Median | IQR |  | *P* value^c^ |  |
| Female | Change from baseline | 3 |  | 8 | 8.6 | 11.3 |  | 9 | 10.2 | 7.2 |  | 0.27 | 0.03; 0.52 | 0.034^a^* |  | 10.9 | 5.1; 14.5 | | 12.3 | 10.3; 15.7 | | 0.491 |  |
|  |  | 6 |  | 8 | 8.0 | 14.9 |  | 9 | 9.9 | 10.7 |  |  |  |  |  | 12.9 | 7.7; 14.3 | | 8.4 | 4.0; 18.2 | | 1.000 |  |
|  |  | 18 |  | 8 | 5.6 | 13.0 |  | 9 | 9.2 | 7.4 |  |  |  |  |  | 10.6 | 3.2; 15.2 | | 9.8 | 8.2; 13.9 | | 0.950 |  |
|  |  | 30 |  | 7 | 0.6 | 14.5 |  | 8 | 10.8 | 11.5 |  |  |  |  |  | 8.3 | -3.9; 13.0 | | 13.4 | 10.1; 15.1 | | 0.228 |  |
|  |  | 42 |  | 6 | -0.6 | 15.9 |  | 8 | 8.5 | 12.8 |  |  |  |  |  | 4.1 | -3.5; 10.0 | | 10.7 | 7.7; 15.9 | | 0.228 |  |
|  | Raw value | 0 |  | 8 | 53.9 | 8.7 |  | 9 | 54.7 | 9.0 |  | 0.27 | 0.03; 0.52 | 0.034^a^* |  | 54.9 | 45.8; 58.9 | | 55.2 | 46.8; 61.2 | | 0.852 |  |
|  |  | 3 |  | 8 | 62.5 | 11.3 |  | 9 | 64.9 | 7.2 |  |  |  |  |  | 63.9 | 58.1; 67.5 | | 66.7 | 64.7; 70.0 | | 0.414 |  |
|  |  | 6 |  | 8 | 61.9 | 14.9 |  | 9 | 64.6 | 10.7 |  |  |  |  |  | 65.9 | 60.7; 67.3 | | 62.8 | 58.4; 72.6 | | 0.852 |  |
|  |  | 18 |  | 8 | 59.5 | 13.0 |  | 9 | 63.9 | 7.4 |  |  |  |  |  | 63.6 | 56.2; 68.2 | | 64.2 | 62.5; 68.2 | | 0.852 |  |
|  |  | 30 |  | 7 | 54.5 | 14.5 |  | 8 | 65.5 | 11.5 |  |  |  |  |  | 61.3 | 49.2; 66.0 | | 67.8 | 64.4; 69.5 | | 0.181 |  |
|  |  | 42 |  | 6 | 53.3 | 15.9 |  | 8 | 63.2 | 12.8 |  |  |  |  |  | 57.1 | 49.5; 63.1 | | 65.1 | 62.1; 70.3 | | 0.181 |  |
|  |  |  |  | Placebo | | |  | GLR | | |  | Effect of GLR^a, b^ | |  |  | Placebo (n = 3) | |  | GLR (n = 2) | |  | Effect of GLR^c^ |  |
|  |  |  |  | n | Mean | SD |  | n | Mean | SD |  | Std.β | 95% CI | *P* value^a, b^ |  | Median | IQR |  | Median | IQR |  | *P* value^c^ |  |
| Male | Change from baseline | 3 |  | 4 | 7.3 | 24.1 |  | 4 | 9.1 | 5.3 |  | 0.52 | 0.08; 1.02 | 0.045^a^* |  | 10.9 | 8.7; 14.2 | | 8.0 | 5.3; 10.8 | | 0.800 |  |
|  |  | 6 |  | 4 | 22.6 | 4.2 |  | 4 | 13.7 | 14.8 |  |  |  |  |  | 15.9 | 14.8; 18.1 | | 19.8 | 16.9; 22.6 | | 0.800 |  |
|  |  | 18 |  | 4 | 5.8 | 19.7 |  | 3 | 22.5 | 9.0 |  |  |  |  |  | 8.7 | 7.9; 8.7 | | 24.1 | 23.8; 24.5 | | 0.200 |  |
|  |  | 30 |  | 3 | 16.0 | 4.9 |  | 2 | 23.5 | 5.5 |  |  |  |  |  | 10.8 | 6.9; 11.4 | | 19.9 | 18.0; 21.9 | | 0.200 |  |
|  |  | 42 |  | 3 | 17.4 | 4.8 |  | 2 | 25.4 | 3.1 |  |  |  |  |  | 7.3 | 7.1; 11.4 | | 21.8 | 20.8; 22.9 | | 0.200 |  |
|  | Raw value | 0 |  | 4 | 40.0 | 16.8 |  | 4 | 51.8 | 14.2 |  | 0.32 | -0.09; 0.73 | 0.136^b^ |  | 51.8 | 44.2; 52.9 | | 55.4 | 50.5; 60.3 | | 0.800 |  |
|  |  | 3 |  | 4 | 47.3 | 24.1 |  | 4 | 60.9 | 5.3 |  |  |  |  |  | 58.4 | 56.2; 61.7 | | 63.4 | 60.7; 66.1 | | 0.800 |  |
|  |  | 6 |  | 4 | 62.6 | 4.2 |  | 4 | 65.5 | 14.8 |  |  |  |  |  | 63.4 | 62.3; 65.6 | | 75.1 | 72.3; 78.0 | | 0.200 |  |
|  |  | 18 |  | 4 | 45.8 | 19.7 |  | 3 | 74.3 | 9.0 |  |  |  |  |  | 56.2 | 55.4; 56.2 | | 79.5 | 79.1; 79.8 | | 0.200 |  |
|  |  | 30 |  | 3 | 56.0 | 4.9 |  | 2 | 75.3 | 5.5 |  |  |  |  |  | 58.3 | 54.4; 58.8 | | 75.3 | 73.3; 77.2 | | 0.200 |  |
|  |  | 42 |  | 3 | 57.4 | 4.8 |  | 2 | 77.2 | 3.1 |  |  |  |  |  | 54.8 | 54.6; 58.9 | | 77.2 | 76.1; 78.3 | | 0.200 |  |
| GLR, Glucoraphanin; FAS, Full-Analysis-Set; PPS, Per-Protocol-Set; IQR; interquartile range showed as “1Q; 3Q”.  Standardized β (Std. β) value, 95% Confidence Interval (95% CI) and *p* value were indicated as the group-time point interaction in multivariate analysis.  ^a^ Multivariate analysis with FAS to confirm the effect for the mean difference throughout the entire study period was performed by a linear mixed model fitted to the following covariates: random effects for each individual; and fixed effects for categorical variables including group and group by time-point interactions; and fixed effects for continuous variables including baseline values, time-point and baseline values by time-point interactions  ^b^ Multivariate analysis with FAS to confirm the effect for the raw value throughout the entire study period was performed by a linear mixed model fitted to the following covariates: random effects for each individual; and fixed effects for categorical variables including group, and group by time-point interactions, continuous variables including time-point.  ^c^ The data for each time-point was analyzed with PPS using the Mann-Whitney U test.  *p* < 0.1^†^, < 0.05*. | | | | | | | | | | | | | | | | | | | | | | | |
